# Evaluation of the variations of mandibular molars and the distance from root apex to the inferior alveolar nerve in Saudi Sub-population: Three-dimensional radiographic evaluation

**DOI:** 10.1371/journal.pone.0317053

**Published:** 2025-02-19

**Authors:** Tariq Mohammed Aqili, Esam Sami Almuzaini, Abdulbari Saleh Aljohani, Ahmed Khaled Al Saeedi, Hassan Abdulmuti Hammudah, Muath Alassaf, Muhannad M. Hakeem

**Affiliations:** 1 Restorative Sciences Department (Endodontic), Taibah University, Madinah, Saudi Arabia; 2 Dental School, Taibah University, Madinah, Saudi Arabia; University of Puthisastra, CAMBODIA

## Abstract

**Aim:**

To investigate the prevalence of various morphological variations in the roots and canals of lower mandibular molar teeth in the Saudi subpopulation and measure the distance from the root apices to the inferior alveolar canal (IAC).

**Materials and methods:**

A cross-sectional analysis was conducted on 149 CBCT scans from Taibah University the College of Dentistry (TUCD). Three evaluators independently reviewed scans for anatomical features such as the number of canals, the presence of radix molaris (RM), and root-to-IANC distances. Teeth observed from the medullary cavity to the root apical layers on the coronal, sagittal and cross-section views. Data was analyzed using SPSS 21.0 software. Statistically significant differences were defined at p < 0.05.

**Results:**

The prevalence of RM ranged between 0.7%-3.4% in lower first and second molars. The number of the canals in the apex ranged between 2–4 canals, with most molars showing three canals. The prevalence of 2 canals in lower first molars is around 2% and in lower second molars is 9.2%. A significant age-related correlation was noted in distances from the mesial and distal roots to the IAC, with values ranging from 0 to 14.7 mm.

**Conclusion:**

The study reveals diverse root and canal morphologies and varying distances to the IANC within the Saudi subpopulation, emphasizing the necessity for precise preoperative radiographic assessments to optimize endodontic outcomes and reduce procedural risks. Findings suggest the need for further research into these anatomical variations to refine diagnostic and treatment strategies in endodontics, particularly in diverse populations to improve patient outcomes.

## Introduction

Root canal treatment is one of the common dental procedures in dentistry, leans heavily on precise anatomical knowledge of the treated tooth to deliver an optimum treatment for the patients to ensure a high success rate [1, 2]. The anatomical complexity of the root canal, especially in mandibular molars, presents significant challenges that directly influence the effectiveness of root canal treatments and their outcomes [2]. Inaccuracies in diagnosing these anatomical variations can lead to complications such as incomplete eradication of pathology or even treatment failure [3]. This emphasizes the demand for accurate and detailed diagnostic methods to guide successful interventions. Mandibular molars are often affected by variations such as extra roots or canals, commonly referred to as radix molaris, which may complicate standard treatment protocols [1–3]. Conventional radiographic techniques, including periapical and panoramic films, have limitations in identifying these three-dimensional structural complexities. Consequently, these conventional methods may not sufficiently reveal the fine features required for comprehensive planning for root canal treatment and can lead to oversight of essential anatomical features [4]. Other different methods of evaluating the root canal configuration exist such as rendering the teeth transparent by clearing techniques, which are invasive techniques [5], or by anatomical reconstruction of the canal system using micro-computed tomography systems or by using cone-beam computed tomography scanning [4]. The course of the inferior alveolar canal varies between individuals, and the distance to the adjacent structures has its own significance during multiple dental procedures such as dental implant placement, tooth extraction, and both surgical and non-surgical endodontic treatment. Multiple methods have been used to assess the proximity of the inferior alveolar canal to its adjacent structure, especially the root apices of the mandibular molars one of these methods is to use CBCT [5].

With the advent of cone-beam computed tomography (CBCT), endodontic diagnosis has incredibly advanced. CBCT offers superior visualization of tooth structures and their relation with surrounding vital structures in three dimensions, allowing endodontists to accurately assess root canal configurations, the number of canals, and their proximity to the inferior alveolar nerve canal (IANC) [5–7]. This detailed data aids in navigating the anatomical complexities of mandibular molars, facilitating more accurate endodontic planning and execution. The use of CBCT has been particularly transformative in recognising atypical anatomical variations that might not be achievable with traditional radiographs, thus improving the predictability and safety of root canal treatments [8, 9]. Furthermore, the root canal morphology of mandibular molars exhibits significant diversity across different populations. Previous studies have revealed that morphological features can vary significantly, affected by ethnic and genetic factors [10, 11]. This variability proposes the need to conduct localized studies to understand specific anatomical patterns and their implications for dental practice in different regions. Despite this, there have been limited studies focusing on this population’s mandibular molar anatomy using CBCT, leaving a gap in the literature.

The population of Madinah, Saudi Arabia, demonstrates a unique demographic for studying orofacial anatomical variations. Because of the diverse ethnicities residing in this city, it serves as a religious destination for Muslims from all over the world. While some studies have explored anatomical variations among Saudi subpopulations, most studies do not thoroughly assess mandibular molar structures and their relation to IAN using CBCT technology allows for precise three-dimensional visualization of these structures, providing insights that conventional radiographs may miss. Additionally, understanding the proximity of these molar roots to the IANC is vital for avoiding procedural risks during dental procedures like endodontic treatment and surgical interventions to ensure patient safety. By addressing this gap, this study can provide valuable baseline data on mandibular molar root and canal morphology specific to the Saudi subpopulation. This data is important not only for root canal treatments within this demographic but also for informing global practitioners about population-specific morphological variations.

In conclusion, this study primarily aims to investigate the morphological variations in the roots and canals of mandibular molars within a Saudi subpopulation using CBCT, as well as assess their proximity to the inferior alveolar nerve canal (IANC). Insights gained may contribute to optimizing endodontic treatment approaches for this demographic and potentially for similar populations.

## Materials and methods

This retrospective cross-sectional study was conducted in accordance with the ethical standards of the institutional review board at Taibah University the College of Dentistry (TUCD), Madinah, Saudi Arabia (Protocol no. TUCDREC/051022/TAqili). The study utilized cone-beam computed tomographic (CBCT) data collected at the university’s dental facility. To report this cross-sectional study STROBE Statement was used [12].

### Sampling

The database of the College of Dentistry provided a total of 400 CBCT scans, which were randomly selected from adult patients aged 15 and older, collected between June 2015 and October 2023, and assessed by November and December 2023. Scans that displayed full mandibular images without any signs of pathology such as internal or external resorption, open apices, or previous root canal treatments were included. From these, 149 scans were selected based on the inclusion criteria, ensuring a focus on relevant diagnostic quality and completeness. The sample size calculation was performed using G*Power software with a T-test, indicating that a minimum of 45 participants is needed to achieve a medium effect size (dz = 0.5) with a statistical power of 95%.

### CBCT imaging parameters

All CBCT scans were performed using a Carestream CS 9300 system. The scanning parameters were standardized at 90 kVp and 4 mA for medium patients, with an acquisition time of 8 seconds. For larger patients, the settings were adjusted to 90 kVp and 5 mA. When the imaging was focused on the mandible only, the same parameters were applied. All scans were conducted by an experienced technician following the manufacturer’s guidelines to minimize radiation exposure.

### Data collection

Three examiners independently reviewed each CBCT scan to identify and record various anatomical features, including the number of roots and canals, root canal configuration, and the presence of anatomical variants such as radix molaris, the Vertucci classification system was applied to categorize canal configurations. The distance from the root apices to the inferior alveolar nerve canal was meticulously measured. The evaluation of the scans was done by the 3D image software used to reconstruct and measure the image. The root canal system of the first and second permanent mandibular molars were observed from the medullary cavity to the root apical layers on the coronal, sagittal and cross-section views.

Prior to the study commencement, calibration sessions were held for all participating examiners, facilitated by experienced endodontists to standardize the measurement process and improve diagnostic consistency. Ten randomly selected cases were examined by the investigators two times within two-week intervals to check the correlation and ensure high inter- and intra-examiner reliability. Discrepancies between examiners were resolved by consensus by experienced endodontists. Inter- and intra-examiner reliability tests were conducted among the three examiners on 10 CBCT scans, yielding values of 93% and 90%, respectively, indicating a high level of reliability.

### Data analysis

SPSS 21.0 software (SPSS, Inc., Chicago, IL, USA) was used for conducting statistical analysis. Descriptive statistics was used to describe the distributions of roots, canals, and their related measurements, as well as the detection rate of root canal morphology. The number of roots and canals, root canal morphology and detection rate of the middle mesial canal (MMC) and radix (R) in different groups according to sex and their bilateral symmetries were analyzed using the Chi-square test. The significance of the correlation between age and the measured distances was determined using the Spearman correlation coefficient, with a significance level set at p < 0.05.

## Results

**Fig 1:**
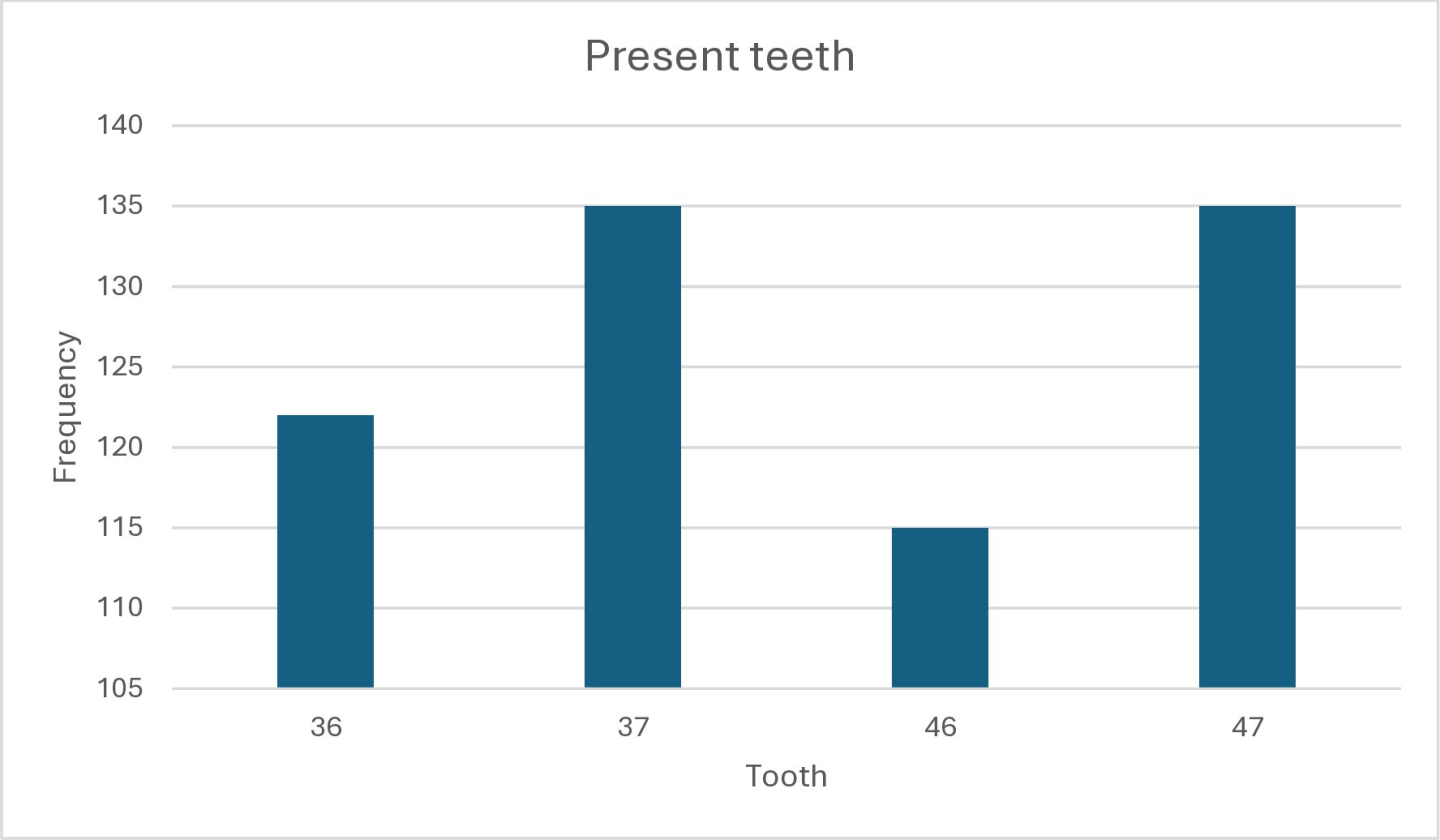
Distribution of tooth types (36, 37, 46, and 47) among 149 CBCT scans.

From an initial pool of 400 CBCT scans, only 149 were eligible to be included in this study. comprising 507 mandibular molars from patients aged 20 to 79 years with a mean age of 39.4 ± 11.7. The distribution of tooth types (36, 37, 46, and 47) across the sample is shown in Fig 1.

**Fig 2:**
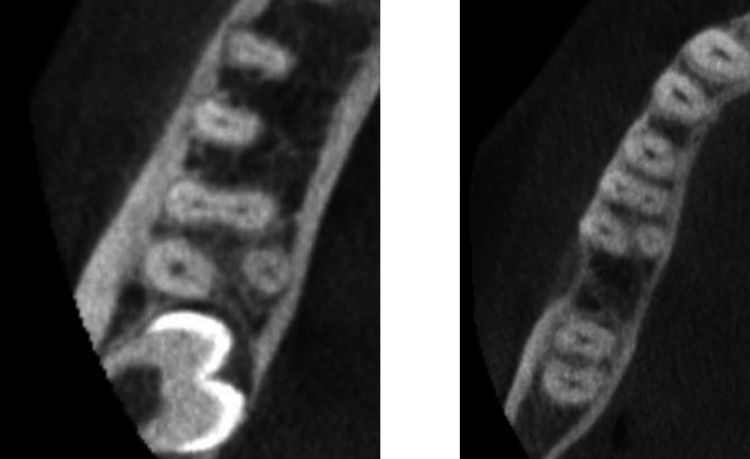
Example of cases with RM.

The study found that *radix molaris* was present in 3.94% of mandibular molars (20 molars), with *radix entomolaris* observed in 3.1% of cases and *radix paramolaris* in 0.8%. The prevalence of these anatomical variations is outlined in Table 1, with the highest occurrence noted in lower right second molars (47). An example of *detected radix molaris* in CBCT is shown in Fig 2.

**Table 1:** Prevalence of *radix molaris (radix entomolaris* and *radix paramolaris)* in mandibular molars.

**Present Roots**	**36**	**37**	**46**	**47**
**Radix Entomolaris**	**(n = 149)**	**(n = 149)**	**(n = 149)**	**(n = 149)**
Not present	145 (97.3%)	146 (98%)	145 (97.3%)	144 (96.6%)
Present	4 (2.7%)	3 (2%)	4 (2.7%)	5 (3.4%)
**Radix Paramolaris**	**(n = 149)**	**(n = 149)**	**(n = 149)**	**(n = 149)**
Not present	149 (100%)	148 (99.3%)	148 (99.3%)	147 (98.7%)
Present	0 (0%)	1 (0.7%)	1 (0.7%)	2 (1.3%)

Detailed distributions of root presence and canal numbers are presented in Tables 1 and 2. The prevalence of radix entomolaris (RE) and radix paramolaris (RP) variants were most frequently identified in the lower right second molars. An example of *detected radix molaris* in CBCT is shown in Fig 2. No significant differences were observed in the prevalence of radix types across the various tooth types. The majority of molars exhibited three canals, although the canals number varied from two to four canals depending on the tooth type.

**Table 2:** Distribution of canal numbers in mandibular molar types (36, 37, 46, and 47).

	36	37	46	47
**Number of canals**	**(n = 114)**	**(n = 126)**	**(n = 101)**	**(n = 122)**
2	2 (1.8%)	11 (8.7%)	2 (2%)	12 (9.8%)
3	96 (84.2%)	112 (88.9%)	3 (89.1%)	106 (86.9%)
4	15 (13.2%)	3 (2.4%)	9 (8.9%)	4 (3.3%)
5	1 (0.9%)	0 (0%)	0 (0%)	0 (0%)

**Fig 3:**
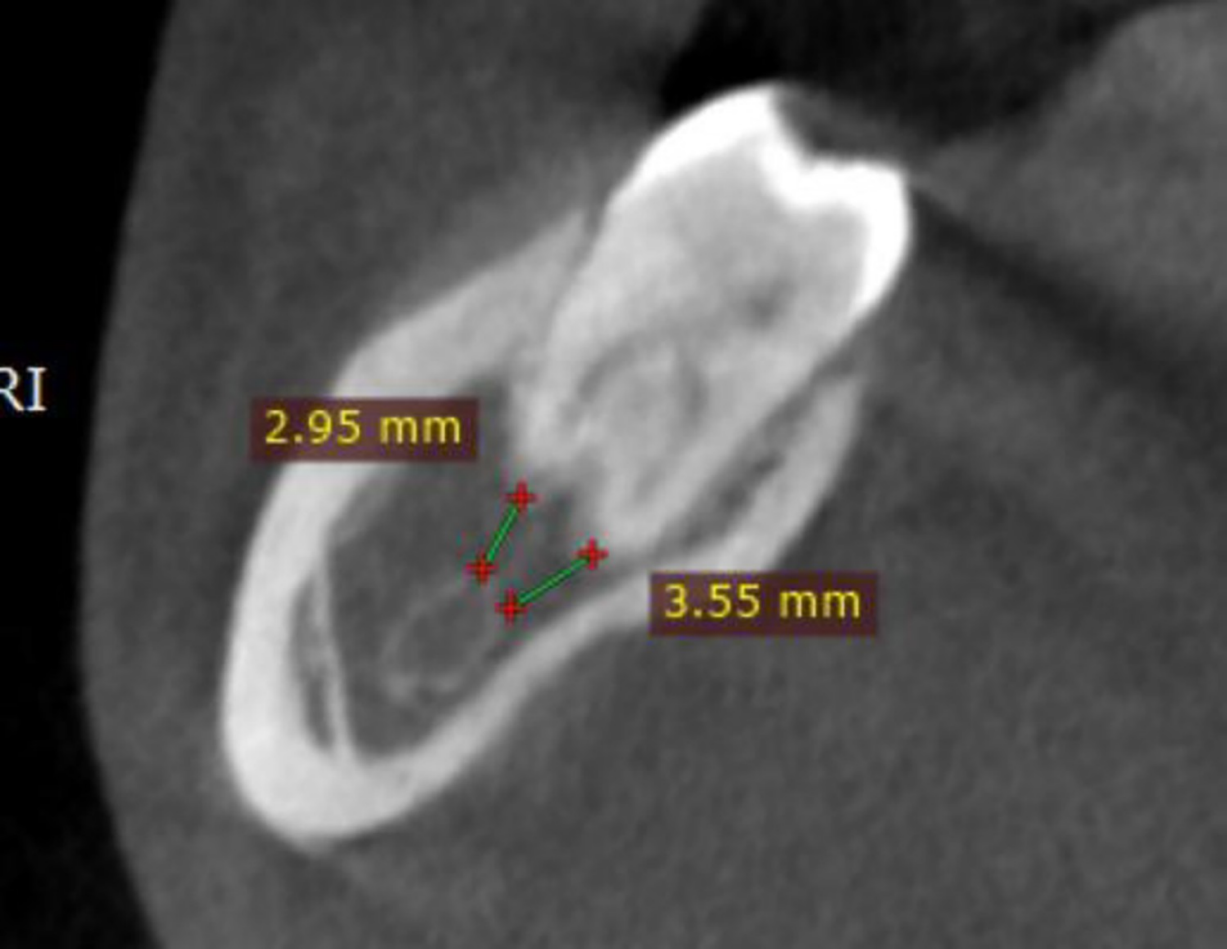
An example of the measurement of the distance from the root end to the inferior alveolar canal.

The distance from the root apices to the inferior alveolar nerve canal (IANC) varied considerably across molars, ranging from 0.0 to 14.7 mm. Age was positively correlated with these distances, particularly for the second molars. Table 3 summarizes the measurements of IANC proximity for each tooth type, providing mean, standard deviation, and range values. Fig 3 shows an example of measuring the distance from the root end to the inferior alveolar canal.

**Table 3:** Distance from root apex to Inferior Alveolar Nerve Canal (IANC) across molar types.

Distance from IANC (mm)	36	37	46	47
**Mesial root**	**(n = 149)**	**(n = 149)**	**(n = 149)**	**(n = 149)**
Mean ± SD.	1.5 ± 2.8	1.7 ± 2.6	1 ± 1.2	1.6 ± 2.5
Median (Min.–Max.)	0 (0–10.4)	0 (0–10.8)	0 (0–14.7)	0 (0–9.3)
**Distal root**	**(n = 149)**	**(n = 149)**	**(n = 149)**	**(n = 149)**
Mean ± SD.	1.5 ± 2.6	1.3 ± 2.2	1.18 ± 2.4	1.4 ± 2.3
Median (Min.–Max.)	0 (0–9.4)	0 (0–8.2)	0 (0–9.3)	0 (0–9.3)
**Radix Entomolaris**	**(n = 149)**	**(n = 149)**	**(n = 149)**	**(n = 149)**
Mean ± SD.	0.09 ± 0.86	0.10 ± 0.88	0.06 ± 0.79	0 ± 0
Median (Min.–Max.)	0 (0–9.8)	0 (0–8.9)	0 (0–9.6)	0 (0–0.0)
**Radix Paramolaris**	**(n = 149)**	**(n = 149)**	**(n = 149)**	**(n = 149)**
Mean ± SD.	0.03 ± 0.34	0.03 ± 0.40	0 ± 0	0.02 ± 0.27
Median (Min.–Max.)	0 (0–4.1)	0 (0–4.9)	0 (0–0)	0 (0–3.4)

Table 4 presents the correlation between patient age and the measured distance from the root apices to the IANC for each molar type using the Spearman correlation coefficient. A statistically significant positive correlation was observed for teeth #37 and #47, indicating that as age increased, the distance from the roots to the IANC also increased for these molars. This relationship, however, was not significant for the lower first molars or for *radix* in general.

**Table 4:** Correlation between patient age and distance from root apices to Inferior Alveolar Nerve Canal (IANC) for mandibular molar types.

Distance from IANC (mm)	Age	
	r_s_	p
**M**		
36	0.085	0.300
37	0.216*	0.008*
46	0.081	0.324
47	0.208*	0.011*
**D**		
36	0.085	0.302
37	0.235*	0.004*
46	0.078	0.341
47	0.225*	0.006*
**RE**		
36	0.136	0.098
37	0.110	0.183
46	0.054	0.517
47	–	–
**PR**		
36	0.139	0.092
37	-0.009	0.917
46	–	–
47	-0.029	0.728

**r**_**s**_ **: Spearman coefficient**

*: Statistically significant at p ≤ 0.05

## Discussion

Understanding the morphology, and complexity of the root canal system is an essential step in estimating the outcome of endodontic treatment and improving their prognosis. A missed root canal can lead to maintaining infected pulp tissue, and act as a reservoir for microorganisms and their byproducts) [13]. This study aims to investigate the morphological variations in mandibular molar roots and canals and their proximity to the inferior alveolar nerve canal (IANC) in a Saudi subpopulation using CBCT technology. The findings reveal a significant prevalence of anatomical variations, notably the presence of *radix molaris* and varied canal configurations. Such variations highlight the importance of CBCT imaging in accurately assessing root canal morphology, which can directly impact clinical decision-making and treatment outcomes. Clinicians should be aware of these anatomical variations, as they may increase the complexity of endodontic procedures and the risk of complications.

This study finds that the radix molaris prevalence in Madinah population (3.97%) aligns with the studies performed in Saudi Arabia [1, 14–17], but when compared globally it moved to the lower end [2, 8, 9, 18–25], especially with East Asian populations where the prevalence is as high as 26% [19]. Our findings align with findings from other regions but show obvious discrepancies in actual prevalence rates. Duman et al. (2019) reported a prevalence of radix entomolaris of 2.9% among patients [6] and 1.2% in teeth in a Turkish cohort [24], illustrating a similar yet distinct anatomical variation compared to neighbouring populations. In contrast, a higher prevalence of 6% was observed in Iranian first molars by Kuzekanani et al. (2017) [26], suggesting a pronounced regional variability that could be attributed to genetic, environmental, or methodological factors. In this study, if radix molaris is found in one of the lower molars, it is not necessarily to be found in the same tooth on the other side in all patients, which agreed with Al-Alawai et al study. Additionally, the highest prevalence of radix molaris was noticed in the lower right second molars. The clinical significance of not trying to find or missing radix molaris can be the Implications of a missed root canal which can lead to maintaining the infection and the need for secondary root canal treatment [13]. However, in the case of finding and treating the radix, the wall that is present on the radix side (lingual or buccal wall) could become weak due to the over-preparation trying to find the radix, making the wall more prone to fracture. So, a careful and conservative approach is mandatory to avoid compromising the tooth structure.

Regarding the number of canals in both first and second molars, the results showed a higher prevalence of roots with 3 canals which agreed with the results of Vertucci’s study [27]. Consistent with global research trends, in the current study, the CBCT was used as a diagnostic modality. This imaging technology has proven invaluable across studies as it is an accurate, three-dimensional, non-invasive method, showing a greater ability to detect morphological features [28]. The CBCT scans that were used in this study were large-volume scans. Therefore, they are less accurate than micro-CT in detecting the apical anatomy and measuring the distances between the anatomical landmarks [29]. This could be one of the weaknesses of this study. The clinical significance of studying the root canal anatomy can help in establishing a baseline of data. Additionally, the variability in canal configurations, particularly the prevalence of three-canal formations and *radix* variants, reinforces the need for precise preoperative imaging to tailor treatment plans to individual anatomical differences. Incorporating CBCT into routine diagnostic protocols, particularly in cases involving complex anatomy, can improve treatment accuracy and reduce complications.

The distance from the end of mesial and distal roots to the inferior alveolar canal increased with age. This could be due to the coronal movement of 2nd molars and bone deposition throughout the patients’ age [30]. In contrast to the findings by Aljarbou et al., which reported distances between the apices of the first and second mandibular molar roots and the inferior alveolar canal ranging from 1.68 to 4.79 mm [5], our study observed greater distances range (0 to 14.7 mm). This discrepancy can likely be attributed to the larger sample size in our research, which was three times that of the Aljarbou et al. study. The clinical implications of these findings are significant. The proximity of mandibular molar roots to the IANC, for example, underscores the importance of detailed imaging to minimize the risk of nerve injury during procedures such as root canal therapy, extractions, and implant placements.

This study does have limitations. The sample was drawn from a single institution, which may limit the generalizability of the results to the broader Saudi population. Furthermore, while CBCT provides valuable three-dimensional imaging, it lacks the ultra-high resolution of micro-CT. Future studies incorporating larger, more diverse samples and higher-resolution imaging are necessary to validate these findings across a wider demographic. Second, this study is a cross-sectional study; the analysis provides a snapshot in time, which limits the capability to draw conclusions about the change in distance from the end of roots to the inferior alveolar canal over time. Longitudinal studies would be required to understand the dynamics of these variations.

Despite these limitations, the study has notable strengths. The use of CBCT allows for a more detailed assessment of root and canal morphology compared to conventional radiographs, offering insights that enhance clinical understanding in endodontics. Additionally, by focusing on a Saudi subpopulation, this study fills a critical gap in the literature and provides data that can guide clinical practices tailored to this demographic. The standardized imaging protocols and robust methodology used in this research also enhance its reproducibility, allowing similar studies to be conducted in other populations for comparative purposes.

## Conclusion

In conclusion, the findings of this study confirm the diversity of root and canal morphology in mandibular molars within the Saudi subpopulation and highlight the importance of advanced imaging techniques like CBCT in endodontic diagnosis and treatment planning. These variations in root and canal anatomy and proximity to the IANC have critical clinical implications, specifically in minimizing treatment errors and enhancing patient safety. To improve endodontic outcomes, clinicians should consider using CBCT for detailed preoperative assessments in cases involving complex anatomy. Expanding these findings to guide clinical policy and practices could support more individualized and safer approaches in endodontic and surgical procedures in Saudi Arabia and similar populations.

## Supporting information

S1 Data Anonymous data master sheet. The file contains anonymized data on mandibular molar evaluations, including patient demographics, tooth presence, root and canal details, and measurements like the distance to the inferior alveolar nerve canal.
(XLSX)
